# Chest X-ray findings of COVID-19 pneumonia in children: Experiences in a multicenter study in Thailand

**DOI:** 10.1371/journal.pone.0309110

**Published:** 2024-09-30

**Authors:** Aunya Kulbun, Prakarn Tovichien, Chanapai Chaiyakulsil, Araya Satdhabudha, Harutai Kamalaporn, Kanokkarn Sunkonkit, Rattapon Uppala, Watit Niyomkarn, Vasinee Norasettekul, Kanokpan Ruangnapa, Chutima Smathakanee, Bararee Choursamran, Rasintra Jaroenying, Tidarat Sriboonyong, Phanthila Sitthikarnkha, Koonkoaw Roekworachai, Thanyarat Ratanavongkosol, Chutima Thongnual, Jitladda Deerojanawong

**Affiliations:** 1 Faculty of Medicine, Department of Pediatrics, Her Royal Highness Maha Chakri Sirindhorn Medical Center, Srinakharinwirot University, Nakhon Nayok, Thailand; 2 Faculty of Medicine Siriraj Hospital, Department of Pediatrics, Division of Pulmonology, Mahidol University, Bangkok, Thailand; 3 Faculty of Medicine, Department of Pediatrics, Thammasat University Hospital, Thammasat University, Pathumthani, Thailand; 4 Center of Excellence in Applied Epidemiology, Thammasat University, Pathumtani, Thailand; 5 Faculty of Medicine, Department of Pediatrics, Ramathibodi Hospital, Mahidol University, Bangkok, Thailand; 6 Faculty of Medicine, Department of Pediatrics, Chiang Mai University, Chiang Mai, Thailand; 7 Faculty of Medicine, Department of Pediatrics, Khon Kaen University, Khon Kaen, Thailand; 8 Faculty of Medicine, Department of Pediatrics, Chulalongkorn University, Bangkok, Thailand; 9 Department of Pediatrics, Charoenkrung Pracharak Hospital, Bangkok, Thailand; 10 Faculty of Medicine, Department of Pediatrics, Prince of Songkla University, Songkhla, Thailand; 11 Department of Pediatrics, Hat Yai Hospital, Songkhla, Thailand; 12 Department of Pediatrics, Sawanpracharak Hospital, Nakhon Sawan, Thailand; 13 Faculty of Medicine, Department of Pediatrics, Phramongkutklao Hospital, Bangkok, Thailand; 14 Department of Pediatrics, Nakornping Hospital, Chiangmai, Thailand; Kaohsuing Medical University Hospital, TAIWAN

## Abstract

**Introduction:**

Although chest X-ray is commonly used to diagnose COVID-19 pneumonia, few studies have explored findings in pediatric patients. This study aimed to reveal chest X-ray characteristics in children with COVID-19 pneumonia and compare between non-severe and severe cases.

**Methods:**

This multicenter, nationwide retrospective study included all children aged 0 to 15 years who were admitted to 13 medical facilities throughout Thailand with COVID-19 pneumonia between January 2020 and October 2021. We analyzed the demographics, clinical features, and chest X-ray results of these children, and compared differences between the non-severe and severe groups.

**Results:**

During the study period, 1018 children (52% male, median age 5 years) were admitted with COVID-19 pneumonia. Most chest radiographic findings showed bilateral (51%) patchy/ground glass opacities (61%) in the central area (64%). Only 12% of the children exhibited typical classification for COVID-19 pneumonia, whereas 74% of chest radiographs were categorized as indeterminate. Comorbidities including chronic lung diseases [adjusted OR (95%CI): 14.56 (3.80–55.75), P-value <0.001], cardiovascular diseases [adjusted OR (95%CI): 7.54 (1.44–39.48), P-value 0.017], genetic diseases [adjusted OR (95%CI): 28.39 (4.55–177.23), P-value <0.001], clinical dyspnea [adjusted OR (95%CI): 12.13 (5.94–24.77), P-value <0.001], tachypnea [adjusted OR (95%CI): 3.92 (1.79–8.55), P-value 0.001], and bilateral chest X-ray infiltrations [adjusted OR (95%CI): 1.99 (1.05–3.78), P-value 0.036] were factors associated with severe COVID-19 pneumonia.

**Conclusion:**

Most children with COVID-19 pneumonia had indeterminate chest X-rays according to the previous classification. We suggest using chest X-rays in conjunction with clinical presentation to screen high-risk patients for early detection of COVID-19 pneumonia.

## 1. Introduction

Since December 2019, Coronavirus Disease 2019 (COVID-19) has been identified. It was discovered in Wuhan, Hubei Province, China, and quickly spread globally. A few months later, the World Health Organization (WHO) declared a global pandemic [1]. Coronaviruses, part of the Coronaviridae family, are viruses with an envelope, positive-sense, single-stranded ribonucleic acid (RNA), and non-segmented structure [2]. These viruses have a wide distribution in both humans and mammals. Of the six previously identified coronaviruses, four cause mild common cold symptoms. Two strains caused Severe Acute Respiratory Syndrome (SARS) in southern China in 2003, and Middle East Respiratory Syndrome (MERS) in Saudi Arabia in 2012 [3].

Children often have more severe viral respiratory infections, leading to hospital visits, hospitalization, and mortality globally. It is unusual for adults to have more severe COVID-19 pneumonia than children. While COVID-19 pneumonia is usually less severe in children, some previously healthy children still face severe and potentially fatal consequences. Among the 4.4 million COVID-19 deaths reported in the MPIDR COVerAGE database, over 17,400 deaths (0.4%) occurred in children and adolescents under 20 years of age. Of those, 53% were among adolescents aged 10–19, and 47% were among children aged 0–9 [4]. As a result, the age-dependent and unique COVID-19 pneumonia patterns in infants, kids, and teenagers highlight the need for further investigation, especially in the early diagnosis and management of COVID-19 pneumonia. Considering these unique patterns, the early diagnosis and management of COVID-19 pneumonia heavily depend on chest X-rays.

Foust et al. published an Expert Consensus Statement on Chest Imaging in Pediatric COVID-19 Patients [5], offering valuable insights into diagnosing pediatric cases. Building on their expertise, the article relies on expert insights, recognizing their importance without comprehensive imaging data. Although the references focus on adult cases, they are essential for understanding chest imaging in pediatric COVID-19 patients due to limited available data for children at that time. With the upcoming COVID-19 outbreak, we review chest X-ray findings in pediatric COVID-19 pneumonia to enhance guidance for managing cases in children. Closing this gap is crucial for achieving early detection of probable COVID-19 pneumonia from chest X-rays in children, distinguishing it from viral bronchiolitis, and gaining unique insights into its radiographic pattern across age groups. This study aims to reveal chest X-ray characteristics in children with COVID-19 pneumonia, comparing findings between non-severe and severe cases, as well as between the Delta and pre-Delta periods, to contribute to understanding unique pneumonia patterns.

## 2. Materials and methods

### 2.1 Study design and subjects

We conducted a nationwide study of pediatric patients aged 0 to 15 years with PCR-confirmed COVID-19 pneumonia admitted at 13 hospitals across Thailand between January 2020 and October 2021. COVID-19 pneumonia is defined by suggestive symptoms, a positive PCR for SARS-CoV-2 RNA, and abnormal chest radiographs [6]. Children with perinatal COVID-19 infection were excluded from the analysis. The Central Research Ethics Council (CREC) of Thailand approved the study (CREC002/2022). Due to the study’s retrospective nature, the informed consent was waived by the CREC. We accessed an online database for research proposes on 23 May 2022 after the approval.

### 2.2 Data collection

Using REDCap (Research Electronic Data Capture), a standardized online database was created with Thammasat University serving as the primary coordination center. Pediatric pulmonologists entered demographic and clinical data, as well as chest X-ray findings obtained through manual chart review, into REDCap. Chest X-ray findings were classified into four types by the consensus of 2 pediatric radiologists and 1 pediatric pulmonologist who were blinded to clinical outcomes and each other radiographic interpretations, following the guidelines in the International Expert Consensus Statement on Chest Imaging in Pediatric COVID-19 Patient Management [7].

1. The typical pattern is characterized by bilateral peripheral and/or subpleural ground-glass opacity and consolidation.
2. The indeterminate pattern exhibits the following features:
  - Unilateral peripheral or peripheral and central ground-glass opacity and/or consolidation.
  - Bilateral peri bronchial thickening and/or opacities.
  - Multifocal or diffuse ground-glass opacity and/or consolidation without specific distribution.
3. The atypical pattern displays the following characteristics:
  - Unilateral segmental or lobar consolidation.
  - Central unilateral or bilateral ground-glass opacity and/or consolidation.
  - Single round consolidation.
  - Presence of pleural effusion or lymphadenopathy.
4. A negative finding means the absence of chest radiographic findings suggestive of pneumonia.

The distribution of lung parenchymal changes was categorized as central, peripheral, or multifocal/ diffuse. Central involvement occurred when the perihilar regions were affected. Peripheral categorization applied when the mid and lateral thirds of the lungs were involved. Multifocal/diffuse classification was used when the involvement was widespread and generalized.

### 2.3 Operational definition

Obesity in children under the age of five was defined as weight-for-height more than three standard deviations (SD) above the World Health Organization (WHO)’s median for child growth [8,9]. Obesity in children and adolescents above the age of five was defined as a BMI-for-age more than 2 SD above the WHO’s median for child growth [8,10].

Immunocompromised children were defined as those who had undergone bone marrow or solid organ transplantation, those currently taking immunosuppressive medications, those undergoing cancer treatment, those with primary immunodeficiencies, and those with advanced or untreated HIV infection [11].

Tachycardia and tachypnea were assessed based on age using WHO guidelines [12]. Desaturation was defined as oxygen saturation from pulse oximetry (SpO2) < 95%.

Those with symptomatic COVID-19 infection and abnormal CXR were considered to have COVID-19 pneumonia. Severity categorization by the National Institutes of Health (NIH) [8] was used to classify pneumonia severity into 2 groups. Severe pneumonia was defined in patients with SpO2 below 94% or critically ill patients requiring respiratory support.

Moreover, COVID-19 pneumonia cases were categorized into pre-Delta (before July 1, 2021) and Delta-dominant (July 1 to October 31, 2021) groups based on thorough nationwide surveillance data from the Ministry of Public Health of Thailand. This classification aids in understanding the evolving COVID-19 landscape during this timeframe.

### 2.4 Statistical analyses

Data were analyzed using STATA for Windows v14.0 (StataCorp LLC, Texas, USA). Clinical features were presented as the median [interquartile range (IQR)] for continuous data and, for categorical variables, as frequency with a percentage. Continuous data were compared using the Wilcoxon rank-sum test. Categorical data were compared using the Chi-square test. A P-value less than 0.05 indicated statistical significance. We used binary logistic regression to identify factors associated with severe COVID-19 pneumonia in univariable and multivariable models. In the multivariable model, a stepwise backward procedure for P-value < 0.05 was used.

## 3. Results

### 3.1 Patients’ characteristics

A total of 1018 children were admitted with confirmed COVID-19 pneumonia during the study period. Fifty-eight children (5.7%) had severe pneumonia requiring oxygen supplementation or respiratory support. Among those, 35 children (60.3%) used low-flow nasal cannula, 3 children (5.2%) used high-flow nasal cannula, 12 children (20.7%) used non-invasive ventilation, and 8 children (13.8%) were intubated and used mechanical ventilator.

Most of the cases were 10–15 years of age (34.9%) and male (52.4%). Eleven children (8.0%) were immunocompromised. One hundred and forty patients (13.8%) had comorbidities. The common comorbidities among the patients included obesity (12.4%), allergic rhinitis (3.4%), asthma (2.8%), hematologic diseases (2.4%), neurologic diseases (1.7%), and chronic lung diseases (1.4%). There was a significantly higher proportion of children with neurologic disease, chronic lung disease, cardiovascular disease, genetic disease, gastrointestinal disease, and oncologic disease in the severe pneumonia group (P-value < 0.05). Most of the patients (82.0%) had a history of COVID-19 contact with family members (Table 1).

**Table 1:** Demographics data of children with COVID-19 pneumonia.

Demographic data		All cases n = 1018	Category by severity		P-value
			Non-severe n = 960	Severe n = 58	
**Age**		5 [2–12]	5 [2–12]	4 [1–11]	0.134
	< 1 year	241 (23.7%)	220 (22.9%)	21 (36.2%)	0.073
	1-<5 years	232 (22.8%)	223 (23.2%)	9 (15.5%)	
	5-<10 years	190 (18.7%)	183 (19.1%)	7 (12.1%)	
	10–15 years	355 (34.9%)	334 (34.8%)	21 (36.2%)	
**Male**		533 (52.4%)	495 (51.6%)	38 (65.5%)	**0.039** *
**Immunocompromise**		11 (8.0%)	7 (6.3%)	4 (16.0%)	0.116
**Comorbidities**		140 (13.8%)	114 (11.9%)	26 (44.8%)	**0.001** *
	Obesity	126 (12.4%)	114 (11.9%))	12 (20.7%)	0.156
	Allergic rhinitis	35 (3.4%)	34 (3.5%)	1 (1.7%)	0.716
	Asthma	29 (2.8%)	26 (2.7%)	3 (5.2%)	0.226
	Hematologic disease	24 (2.4%)	2 (3.4%)	22 (2.3%)	0.643
	Neurologic disease	17 (1.7%)	10 (1.0%)	7 (12.1%)	**0.001** *
	Chronic lung disease	14 (1.4%)	7 (0.7%)	7 (12.1%)	**0.001** *
	Cardiovascular disease	13 (1.3%)	8 (0.8%)	5 (8.6%)	**0.001** *
	Genetic disease	9 (0.9%)	3 (0.3%)	6 (10.3%)	**0.001** *
	Endocrine disease	8 (0.8%)	0 (0%)	8 (0.8%)	1.000
	Developmental disease ^a^	6 (0.6%)	5 (0.5%)	1 (1.7%)	0.297
	Gastrointestinal disease	5 (0.5%)	3 (0.3%)	2 (3.4%)	**0.029** *
	Oncologic disease	4 (0.4%)	2 (0.2%)	2 (3.4%)	**0.018** *
**Index case**					
	Family member	833 (82.0%)	791 (82.6%)	42 (72.4%)	**0.001** *
	Community cluster	19 (1.9%)	15 (1.6%)	4 (6.9%)	
	School/ daycare	9 (0.9%)	9 (0.9%)	0 (0%)	
	Others ^b^	157 (15.4%)	145 (15.1%)	12 (20.7%)	

^a^ Developmental disease included autism and attention deficit hyperactivity disorder.

^b^ Others included neighborhood, hospital, travel, and unknown index case.

Data presented as median [interquartile range] and number (percent).

*P-value < 0.05 indicated statistical significance.

### 3.2 Clinical characteristics

The most frequent symptoms were fever (71.8%), cough (58.6%), and rhinorrhea (37.8%). Gastrointestinal symptoms, including nausea/vomiting/diarrhea, were reported by 12.4% of patients, and 6.5% reported dyspnea. Only 276 patients (27.1%) had a high-grade fever above 38.5 degrees Celsius, and only 71 patients (7.0%) had tachypnea for age.

There was a significantly higher proportion of children with dyspnea in the severe pneumonia group (P-value < 0.001). Moreover, they had a significant proportion with tachypnea and tachycardia for age (P-value < 0.001). However, the cycle threshold of SARs-CoV-2 PCR and white blood cell count in the severe pneumonia group did not significantly differ from the non-severe group (P-value = 0.574 and 0.194, respectively) (Table 2).

**Table 2:** Clinical characteristics of children with COVID-19 pneumonia.

Clinical features		All cases n = 1018	Category by severity		P-value
			Non-severe n = 960	Severe n = 58	
**Symptoms**					
	Fever	731 (71.8%)	685 (71.4%)	46 (79.3%)	0.191
	Cough	597 (58.6%)	557 (58.0%)	40 (69.0%)	0.100
	Rhinorrhea	385 (37.8%)	366 (38.1%)	19 (32.8%)	0.413
	Sore throat	131 (12.9%)	129 (13.4%)	2 (3.4%)	**0.027** *
	Diarrhea	91 (8.9%)	84 (8.8%)	7 (12.1%)	0.390
	Anosmia	75 (7.4%)	74 (7.7%)	1 (1.7%)	0.117
	Dyspnea	66 (6.5%)	40 (4.2%)	26 (44.8%)	**0.001** *
	Headache/vertigo	58 (5.7%)	56 (5.8%)	2 (3.4%)	0.768
	Nasal congestion	47 (4.6%)	45 (4.7%)	2 (3.4%)	1.000
	Vomiting	36 (3.5%)	34 (3.5%)	2 (3.4%)	1.000
	Rash	21 (2.1%)	20 (2.1%)	1 (1.7%)	1.000
	Tasteless	17 (1.7%)	17 (1.8%)	0 (0%)	0.618
**Clinical signs**					
	Temperature > 38.5°C	276 (27.1%)	263 (27.4%)	13 (22.4%)	0.407
	Tachypnea for age	71 (7.0%)	50 (5.2%)	21 (36.2%)	**0.001** *
	Tachycardia for age	41 (4.0%)	29 (3.0%)	12 (20.7%)	**0.001** *
**The cycle threshold of SARs-CoV-2 PCR**		20 [16–25]	20 [16–26]	21 [18–25]	0.574
**White blood cell count** (cells/mm^3^)		7580 [5645–10240]	7740 [5645–10305]	8020 [6145–11665]	0.194

**Abbreviations:** [PCR: Polymerase chain reaction].

Data presented as number (percent) or median [interquartile range].

*P-value < 0.05 indicated statistical significance.

### 3.3 Chest X-ray findings

Symptoms until chest X-ray occurred over a median of 2 [IQR: 1–3] days. According to the chest X-ray categories, only 125 patients had a typical pattern (12.3%). Most (74.0%) showed an indeterminate pattern. However, there was a higher proportion of children with both typical and atypical patterns in the severe pneumonia group.

Normal aeration was observed in 638 patients (62.7%). Only 202 patients (19.8%) had hyperaeration, indicating concomitant lower-airway involvement. Patchy/ ground glass opacities were seen in 622 patients (61.1%). Unilateral consolidation accounted for 49.3%, and bilateral for 50.7%. A predominantly central location was observed in 651 patients (63.9%). Involvement of the peripheral space was seen in only 367 patients (36.1%).

In the severe group, there was a significantly higher proportion with various conditions. These included both typical and atypical pattern, hypoaeration, and bilateral infiltrations (P-value = 0.011, 0.005, and 0.001, respectively) (Table 3).

**Table 3:** Chest radiograph findings categorized by severity.

Clinical features		All cases n = 1018	Category by severity		P-value
			Non-severe n = 960	Severe n = 58	
**Days of symptoms at CXR**		2 [1–3]	2 [1–3]	2 [1–3]	0.580
**CXR categories**					
	Typical	125 (12.3%)	116 (12.1%)	9 (15.5%)	**0.011** *
	Indeterminate	753 (74.0%)	719 (74.9%)	34 (58.6%)	
	Atypical	140 (13.8%)	125 (13.0%)	15 (25.9%)	
**Aeration**					
	Normoaeration	638 (62.7%)	595 (62.0%)	43 (74.1%)	**0.005** *
	Hyperaeration	202 (19.8%)	200 (20.8%)	2 (3.4%)	
	Hypoaeration	178 (17.5%)	165 (17.2%)	13 (22.4%)	
**Types of infiltration**					
	Patchy/ GGO	622 (61.1%)	588 (61.3%)	34 (58.6%)	0.590
	Interstitial/ reticular	389 (38.2%)	366 (38.1%)	23 (39.7%)	
	Nodular	7 (0.7%)	6 (0.6%)	1 (1.7%)	
**Sides of infiltrations**					
	Unilateral	502 (49.3%)	487 (50.7%)	15 (25.9%)	**0.001** *
	Bilateral	516 (50.7%)	473 (49.3%)	43 (74.2%)	
	Multifocal/ diffuse	70 (6.9%)	63 (6.6%)	7 (12.1%)	0.110
**Sites of infiltrations**					
	Central	651 (63.9%)	607 (63.2%)	44 (75.9%)	0.052
	Peripheral	367 (36.1%)	353 (36.8%)	14 (24.1%)	
**Other findings**					
	Atelectasis	5 (0.5%)	4 (0.4%)	1 (1.7%)	0.400
	Pleural effusion	4 (0.4%)	1 (0.1%)	3 (5.2%)	

Data presented as median [interquartile range] or number (percent).

*P-value < 0.05 indicated statistical significance.

Factors associated with severe COVID-19 pneumonia were analyzed as shown in **Table 4**. Comorbidities including chronic lung diseases [adjusted OR (95%CI): 14.56 (3.80–55.75), P-value <0.001], cardiovascular diseases [adjusted OR (95%CI): 7.54 (1.44–39.48), P-value 0.017], genetic diseases [adjusted OR (95%CI): 28.39 (4.55–177.23), P-value <0.001], clinical dyspnea [adjusted OR (95%CI): 12.13 (5.94–24.77), P-value <0.001], tachypnea [adjusted OR (95%CI): 3.92 (1.79–8.55), P-value 0.001], and bilateral chest X-ray infiltrations [adjusted OR (95%CI): 1.99 (1.05–3.78), P-value 0.036] were factors associated with severe COVID-19 pneumonia.

**Table 4:** Factors associated with severe COVID-19 pneumonia.

Factors		OR (95% CI)	P-value	Adjusted OR (95%CI)	P-value
**Demographic**					
	Male	1.79 (1.02–3.11)	0.041		
	Neurologic diseases	13.04 (4.77–35.66)	<0.001		
	Chronic lung diseases	18.69 (6.32–55.29)	<0.001	14.56 (3.80–55.75)	<0.001
	Cardiovascular diseases	11.23 (3.55–35.49)	<0.001	7.54 (1.44–39.48)	0.017
	Genetic diseases	36.81 (8.95–151.33)	<0.001	28.39 (4.55–177.23)	<0.001
**Clinical characteristics**					
	Dyspnea	18.69 (10.19–32.28)	<0.001	12.13 (5.94–24.77)	<0.001
	Tachypnea	10.33 (5.63–18.94)	<0.001	3.92 (1.79–8.55)	0.001
	Tachycardia	8.38 (4.02–17.47)	<0.001		
**Chest radiograph findings**					
	Bilateral infiltration	2.20 (1.27–3.79)	0.005	1.99 (1.05–3.78)	0.036
	Multifocal/ diffuse infiltration	1.95 (0.85–4.48)	0.114		

*P-value < 0.05 indicated statistical significance.

In comparison between the Delta and pre-Delta periods, a significantly higher proportion of children with typical radiographic findings were found during the Delta period. (13.3% vs 7.0%; P-value = 0.006) (Table 5).

**Table 5:** Chest radiograph findings categorized by COVID-19 strain.

Clinical features		All cases n = 1018	Categorized by strain		P-value
			Pre-delta n = 158	Delta n = 860	
**CXR categories**					
	Typical	125 (12.3%)	11 (7.0%)	114 (13.3%)	0.006*
	Indeterminate	753 (74.0%)	115 (72.8%)	638 (74.2%)	
	Atypical	140 (13.8%)	32 (20.3%)	108 (12.6%)	
**Aeration**					
	Normoaeration	638 (62.7%)	95 (60.1%)	543 (63.1%)	0.601
	Hyperaeration	202 (19.8%)	31 (19.6%)	171 (19.9%)	
	Hypoaeration	178 (17.5%)	32 (20.3%)	146 (17.0%)	
**Types of infiltration**					
	Patchy/ GGO	622 (61.1%)	103 (65.2%)	519 (60.3%)	0.288
	Interstitial/ reticular	389 (38.2%)	53 (33.5%)	336 (39.1%)	
	Nodular	7 (0.7%)	2 (1.3%)	5 (0.6%)	
**Sides of infiltrations**					
	Unilateral	502 (49.3%)	82 (51.9%)	420 (48.8%)	0.241
	Bilateral	516 (50.7%)	76 (48.1%)	440 (51.1%)	
	Multifocal/ diffuse	70 (6.9%)	6 (3.8%)	64 (7.4%)	0.096
**Sites of infiltrations**					
	Central	651 (63.9%)	99 (62.7%)	552 (64.2%)	0.713
	Peripheral	367 (36.1%)	59 (37.3%)	308 (35.8%)	
**Other findings**					
	Atelectasis	5 (0.5%)	3 (1.9%)	2 (0.2%)	1.000
	Pleural effusion	4 (0.4%)	2 (1.3%)	2 (0.2%)	

Data presented as number (percent).

*P-value < 0.05 indicated statistical significance.

## 4. Discussion

This study aimed to examine chest X-rays in 1018 pediatric patients at 13 hospitals who were confirmed COVID-19 pneumonia by PCR. Among these patients, the most frequent symptoms of pneumonia were fever, cough, rhinorrhea, sore throat, and diarrhea. These symptoms were like mild symptomatic COVID-19 infection [13–16]. There was a significantly higher proportion of children with dyspnea in the severe pneumonia group. Additionally, they exhibited tachypnea and tachycardia for their age. Hence, the study emphasizes the need to raise concerns about severe pneumonia and closely monitor children with these findings.

While pneumonia is one of the most serious illnesses for pediatric patients, COVID-19 pneumonia is not as common as in adults due to several reasons including varied adaptive and innate immune responses to COVID-19 [15]. Diagnosing pneumonia primarily through physical examinations is challenging during an outbreak. Chest radiographs aid in diagnosis. However, it is unclear whether we need to surveillance chest radiographs in all children with COVID-19 infection and whether the characteristic COVID-19 pneumonia findings in adults apply to pediatric patients.

In this study, we found that common chest X-ray findings included bilateral (50.7%), patchy/ ground glass opacities (61.1%) in central distribution (63.9%). Chest X-rays were mostly categorized as indeterminate patterns in both non-severe and severe groups. However, the severe groups showed a higher rate of the typical pattern for COVID-19. This pattern is characterized by the bilateral distribution of peripheral and/or subpleural ground glass opacities (GGOs) and/or consolidation [5]. Moreover, severe pneumonia groups exhibited more hypoaeration than the non-severe group. Therefore, the study also emphasizes the need to raise concerns about severe pneumonia and closely monitor children with these findings.

The multicenter study on chest X-ray findings in 81 children with PCR-confirmed COVID-19 pneumonia in Europe identified increased central peribronchovascular markings or perihilar bronchial wall thickening and consolidation as the most frequent abnormal findings, observed in 58% and 35% of patients, respectively. Furthermore, GGOs were found in 19%, and interstitial patterns in only 16% of their patients. Their study revealed more patients with pleural effusion (7%), atelectasis (2%), and cases of pneumothorax (2%)—none of which were observed in our study [15].

The single-center study in India examined chest X-ray findings in 90 hospitalized children with confirmed COVID-19. It revealed bilateral peribronchial cuffing in 68%, consolidation in 11%, bilateral central GGOs in 2%, and unilateral pleural effusion in 1% of patients. Of the 61 patients with peribronchial cuffing, 69% (42/61) had changes centrally, and the remaining 31% (19/61) showed diffuse bilateral peribronchial cuffing. Unilateral peribronchial cuffing was not observed in any of the cases. In the 10 patients with consolidation, 60% (6/10) had bilateral and diffuse parenchymal changes. Another 20% (2/10) showed central distribution, while 10% (1/10) had unilateral and diffuse changes, and the remaining 10% (1/10) displayed bilateral changes with lower zone predominance. Hyperinflation of both lungs was observed in seven patients. In no case was hyperinflation an isolated finding; it was consistently accompanied by parenchymal changes [17].

A single-center study in Spain looked at 44 children with PCR-confirmed COVID-19 pneumonia. It found that peribronchial cuffing was the most common finding (86.3%), followed by GGOs at 50%. In both cases, central distribution was more common than peripheral, like our findings. All cases with peribronchial cuffing were bilateral. Among those with GGOs, 47.7% were bilateral, and 2.3% were unilateral. In both cases, bilateral abnormality was more common, like our findings. Consolidations accounted for 18.1% of the cases, with 11.3% being unilateral and 6.8% being bilateral. Less common findings included pleural effusion (9.1%) [18].

Another single-center study looked at chest X-ray findings in 51 children with PCR-confirmed COVID-19 infection, including multisystem inflammatory syndrome in children (MIS-C), in the USA. The study found that interstitial opacities, similar to our findings, were the most frequent finding, present in 16 of 51 patients (31%). Interstitial opacities were mostly diffuse, noted in 10 of 16 children (63%). Alveolar opacities were observed in 14 of 51 children (27%). The distribution of alveolar opacity included mostly diffuse patterns in 4 of 14 cases, followed by scattered (3 of 14), peripheral (3 of 14), and central (2 of 14). Additionally, pleural effusion was identified in 5 of the 51 cases (10%). Pneumothorax was not present on any chest X-ray, similar to our findings [19].

Chest X-ray images in children with COVID pneumonia mimic viral pneumonia spreading from the upper to the lower respiratory tract via the airways. Therefore, the inflammatory process leads to bronchial wall thickening from swelling and mucus hypersecretion [20]. However, only a few patients showed primarily lower airway involvement, revealing hyperaeration and/or atelectasis in their chest X-ray. In adults, common chest X-ray findings include bilateral ground glass opacities with peripheral distribution. Only a few patients had perihilar interstitial infiltration as pediatric patients [1,21,22].

Because pulmonary changes in adults with COVID-19 pneumonia are mostly found peripherally in the lower lobes, lung ultrasound helps detect pulmonary pathology abutting the pleura, especially in the intensive care unit [23]. However, the application of lung ultrasound in children with COVID-19 pneumonia has limitations. Even though pediatric lung ultrasound provides better image quality than in adults, thanks to a thinner chest wall, our research reveals that pulmonary changes in children with COVID-19 pneumonia are predominantly located centrally.

This study also supports the recommendation by finding that comorbidities, including chronic lung diseases, cardiovascular diseases, and genetic diseases were factors associated with severe COVID-19 pneumonia. Similarly, Sarkar et al., like our study, discovered that 50% of patients with severe pneumonia had related comorbidities, such as congenital heart disease, neurological conditions, and childhood cancer [24]. Furthermore, our research group has previously identified 4 key pneumonia risk factors in children with COVID-19: age < 5 years, number of comorbidities, fever, and dyspnea. These findings are crucial for early detection and management. Leveraging our predictive score for pediatric COVID-19 pneumonia, outlined in a previous report [25], we recommend using chest X-ray to screen symptomatic patients in the high-risk group and closely monitor patients with dyspnea, and tachypnea for age, especially those whose chest X-ray reveals bilateral abnormalities.

Our study is one of the largest studies revealing the chest X-ray findings of children with COVID-19 pneumonia. In this multicenter study, a standardized online database via RedCap was utilized to collect data from across the country, providing a highly robust database to conduct a study. Furthermore, every institution followed the Thai National Guidance for the diagnosis and treatment of COVID-19 by the Department of Medical Services during the study period. Our study focused on the pre-Delta and Delta-dominant eras of COVID-19 to understand how certain variants might impact chest X-ray results during these critical periods. A significantly higher proportion of children with typical radiographic findings were found during the Delta period. Hence, it’s crucial to explore how different COVID-19 variants might affect chest X-ray results in the future study.

However, it’s crucial to note that the heterogeneous image quality of chest X-rays and the experience of pediatric radiologists and pulmonologists in each institution may impact the accurate detection and interpretation of chest X-ray findings. Furthermore, it’s crucial to consider that children with proven COVID-19 might have concurrent bacterial or viral infections, adding complexity to the interpretation of chest X-ray findings. This study lacks a direct comparison with other respiratory diseases. This limitation hinders assessing the specificity and sensitivity of X-ray manifestations of COVID-19 pneumonia, which merits further investigation. Despite these challenges, our study successfully outlined the spectrum of chest X-rays in children with COVID-19 pneumonia at the national level, providing valuable insights into the field.

## 5. Conclusion

Most children with COVID-19 pneumonia had indeterminate chest X-rays according to the previous classification. Thus, relying solely on a chest X-ray might not be enough to early detect COVID-19 pneumonia in children. To overcome this limitation, we suggest using chest X-rays in conjunction with clinical presentation to screen high-risk patients with symptoms for early detection of COVID-19 pneumonia.

## References

[1] Salehi S, Abedi A, Balakrishnan S, Gholamrezanezhad A. Coronavirus Disease 2019 (COVID-19): A Systematic Review of Imaging Findings in 919 Patients. AJR Am J Roentgenol. 2020;215(1):87–93. doi: 10.2214/AJR.20.23034 32174129

[2] Kooraki S, Hosseiny M, Myers L, Gholamrezanezhad A. Coronavirus (COVID-19) Outbreak: What the Department of Radiology Should Know. J Am Coll Radiol. 2020;17(4):447–51. doi: 10.1016/j.jacr.2020.02.008 32092296 PMC7102595

[3] Yoon SH, Lee KH, Kim JY, Lee YK, Ko H, Kim KH, et al. Chest Radiographic and CT Findings of the 2019 Novel Coronavirus Disease (COVID-19): Analysis of Nine Patients Treated in Korea. Korean J Radiol. 2020;21(4):494–500. doi: 10.3348/kjr.2020.0132 32100485 PMC7082662

[4] United Nations International Children’s Emergency Fund. Child mortality and COVID-19 2023 [28 November 2023]. Available from: https://data.unicef.org/topic/child-survival/covid-19/.

[5] Foust AM, Phillips GS, Chu WC, Daltro P, Das KM, Garcia-Peña P, et al. International Expert Consensus Statement on Chest Imaging in Pediatric COVID-19 Patient Management: Imaging Findings, Imaging Study Reporting, and Imaging Study Recommendations. Radiol Cardiothorac Imaging. 2020;2(2):e200214. doi: 10.1148/ryct.2020200214 33778577 PMC7233446

[6] Parisi GF, Indolfi C, Decimo F, Leonardi S, Miraglia Del Giudice M. COVID-19 Pneumonia in Children: From Etiology to Management. Front Pediatr. 2020;8:616622. doi: 10.3389/fped.2020.616622 33381482 PMC7767924

[7] Desoky SM, Andronikou S, Brody AS, Hirsch FW. Re: "International Expert Consensus Statement on Chest Imaging in Pediatric COVID-19 Patient Management: Imaging Findings, Imaging Study Reporting and Imaging Study Recommendations". Radiol Cardiothorac Imaging. 2020;2(3):e200305. doi: 10.1148/ryct.2020200305 33778593 PMC7325516

[8] COVID-19 Treatment Guidelines Panel. Coronavirus Disease 2019 (COVID-19) Treatment Guidelines: National Institutes of Health; [updated 1 November 2023. Available from: https://www.covid19treatmentguidelines.nih.gov. 34003615

[9] World Health Organization. World Health Organization Child Growth Standards 2006 [Available from: https://www.who.int/tools/child-growth-standards.

[10] de Onis M, Onyango AW, Borghi E, Siyam A, Nishida C, Siekmann J. Development of a WHO growth reference for school-aged children and adolescents. Bull World Health Organ. 2007;85(9):660–7. doi: 10.2471/blt.07.043497 18026621 PMC2636412

[11] Division of Viral Diseases National Center for Immunization and Respiratory Diseases (NCIRD). COVID-19 Vaccines for People who are Moderately or Severely Immunocompromised 2022 [Available from: https://www.cdc.gov/coronavirus/2019-ncov/vaccines/recommendations/immuno.html.

[12] World Health Organization. Guidelines for the management of common illnesses with limited resources. Pocket book of hospital care for children. 2nd ed. Geneva: World Health Organization; 2013. 24006557

[13] Melo MM, Neta MMR, Neto ARS, Carvalho ARB, Magalhães RLB, Valle A, et al. Symptoms of COVID-19 in children. Braz J Med Biol Res. 2022;55:e12038. doi: 10.1590/1414-431X2022e12038 35703681 PMC9200047

[14] Vosoughi F, Makuku R, Tantuoyir MM, Yousefi F, Shobeiri P, Karimi A, et al. A systematic review and meta-analysis of the epidemiological characteristics of COVID-19 in children. BMC Pediatr. 2022;22(1):613. doi: 10.1186/s12887-022-03624-4 36273121 PMC9587668

[15] Ludvigsson JF. Systematic review of COVID-19 in children shows milder cases and a better prognosis than adults. Acta Paediatr. 2020;109(6):1088–95. doi: 10.1111/apa.15270 32202343 PMC7228328

[16] Caro-Dominguez P, Shelmerdine SC, Toso S, Secinaro A, Toma P, Damasio MB, et al. Thoracic imaging of coronavirus disease 2019 (COVID-19) in children: a series of 91 cases. Pediatr Radiol. 2020;50(10):1354–68. doi: 10.1007/s00247-020-04747-5 32749530 PMC7399600

[17] Vendhan K, Kathirrajan M, Kathirvelu G, S B. Chest radiograph findings in children with COVID-19-A retrospective analysis from a tertiary care paediatric hospital in South India. J Trop Pediatr. 2023;69(2). doi: 10.1093/tropej/fmad016 36913556

[18] Oterino Serrano C, Alonso E, Andrés M, Buitrago NM, Pérez Vigara A, Parrón Pajares M, et al. Pediatric chest x-ray in covid-19 infection. Eur J Radiol. 2020;131:109236. doi: 10.1016/j.ejrad.2020.109236 32932176 PMC7448740

[19] Biko DM, Ramirez-Suarez KI, Barrera CA, Banerjee A, Matsubara D, Kaplan SL, et al. Imaging of children with COVID-19: experience from a tertiary children’s hospital in the United States. Pediatr Radiol. 2021;51(2):239–47. doi: 10.1007/s00247-020-04830-x 32945888 PMC7498743

[20] Pierce CA, Preston-Hurlburt P, Dai Y, Aschner CB, Cheshenko N, Galen B, et al. Immune responses to SARS-CoV-2 infection in hospitalized pediatric and adult patients. Sci Transl Med. 2020;12(564). doi: 10.1126/scitranslmed.abd5487 32958614 PMC7658796

[21] Miyashita N, Nakamori Y, Ogata M, Fukuda N, Yamura A, Ishiura Y, et al. Early identification of novel coronavirus (COVID-19) pneumonia using clinical and radiographic findings. J Infect Chemother. 2022;28(5):718–21. doi: 10.1016/j.jiac.2022.02.005 35190258 PMC8828417

[22] Sun Z, Zhang N, Li Y, Xu X. A systematic review of chest imaging findings in COVID-19. Quant Imaging Med Surg. 2020;10(5):1058–79. doi: 10.21037/qims-20-564 32489929 PMC7242306

[23] Buonsenso D, Pata D, Chiaretti A. COVID-19 outbreak: less stethoscope, more ultrasound. Lancet Respir Med. 2020;8(5):e27. doi: 10.1016/S2213-2600(20)30120-X 32203708 PMC7104316

[24] Sarkar M, Das B, Mahapatra MK, Roychowdhoury S, Das S, Konar MC. A Retrospective Analysis of Clinical Manifestations, Management and Outcome of Acute Respiratory Distress Syndrome Associated with Coronavirus Disease-2019 Infection in Children. Indian J Crit Care Med. 2022;26(3):331–8. doi: 10.5005/jp-journals-10071-24145 35519909 PMC9015940

[25] Satdhabudha A, Chaiyakulsil C, Uppala R, Niyomkarn W, Tovichien P, Norasettekul V, et al. Development and validation of the predictive score for pediatric COVID-19 pneumonia: A nationwide, multicenter study. PLoS One. 2022;17(8):e0273842. doi: 10.1371/journal.pone.0273842 36037228 PMC9423652

